# Improving the profiling of wheat bacterial and fungal endophytic communities—a PCR clamping approach

**DOI:** 10.3389/fmicb.2025.1690976

**Published:** 2025-10-31

**Authors:** Benjamin Dubois, Mathieu Delitte, Claude Bragard, Anne Legrève, Anne Chandelier, Frédéric Debode

**Affiliations:** ^1^Bioengineering Unit, Life Sciences Department, Walloon Agricultural Research Centre, Gembloux, Belgium; ^2^Earth and Life Institute – Applied Microbiology, Plant Health, UCLouvain, Louvain-la-Neuve, Belgium; ^3^Plant and Forest Health Unit, Life Sciences Department, Walloon Agricultural Research Centre, Gembloux, Belgium

**Keywords:** clamp, PNA, blocking primer, microbiome, endophyte, wheat, *Triticum aestivum*, metabarcoding

## Abstract

**Background:**

Plant-associated endophytic microbial communities are an important source of biological diversity. To study them, efficient, robust, and standardized characterization methods are necessary. These communities are usually profiled using amplicon high-throughput sequencing (metabarcoding), but the large amount of host DNA often leads to substantial co-amplification of organellar sequences, thereby hampering accurate characterization. A promising solution is the use of PCR clamps, modified oligomers that block non-target DNA amplification. However, no practical guidelines are currently available to support their development, and no sets of clamps enabling comprehensive characterization of endophytic bacterial and fungal communities associated with wheat (*Triticum aestivum ssp. aestivum*) have been reported.

**Results:**

We developed PCR clamps to block wheat DNA co-amplification while targeting bacterial or fungal populations. For bacteria, two clamping strategies [blocking primers and peptide nucleic acid (PNA)] were evaluated on the 16S V5V7 region. The PNA exhibited superior efficiency (99.8% bacterial reads), whereas blocking primers still performed well (67–98%) and offered a cheaper alternative. The PNA approach was retained for subsequent designs due to its higher efficiency, and two additional PNAs targeting the 16S V4 region were designed to block chloroplast and mitochondrial DNA, respectively. The best results were achieved using both PNAs simultaneously, with 80% of reads being of bacterial origin. For fungi, two PNA clamps were designed targeting ITS1 and ITS2, leading to a substantial reduction in wheat DNA co-amplification, with up to 94 and 75% fungal reads obtained using the ITS1- and ITS2-targeting PNA, respectively. The results also highlighted that profiling endophytic communities without clamps risks significantly underestimating microbial diversity. Furthermore, four bacterial and fungal mock communities were created as tools for standardization and internal control, confirming that our clamps do not inhibit microbial DNA amplification.

**Conclusion:**

Whereas amplifications without clamps yielded almost exclusively plant reads, the clamps developed here significantly increased the proportion of microbial reads. This in turn enhanced microbial diversity recovery and the reliability of conclusions drawn from endophytic community analyses. The methodology described provides a framework for clamp development that can be reproduced and adapted to any other host species.

## 1 Introduction

Wheat (*Triticum aestivum*) is a staple crop that feeds a significant portion of the global population, representing more than 27% of the world cereal production in 2023 (FAO, 2024). Given its importance, understanding the factors that influence wheat health and productivity is crucial, especially as agricultural systems face increasing challenges from climate change and pathogen pressures (Savary et al., 2019). Recent research has highlighted the critical role of the endophytic microbiome—microorganisms that live within plant tissues—in promoting plant growth, enhancing nutrient acquisition, and bolstering resistance to pathogens (Jog et al., 2014; Busby et al., 2016; Fadiji and Babalola, 2020; Zhang J. et al., 2021). In wheat, these endophytic communities are particularly valuable for combating major fungal diseases, such as *Zymoseptoria tritici* blotch and *Fusarium* head blight, which can devastate yields (Latz et al., 2020; Rojas et al., 2020). Since they have a significant impact on the plant fitness and productivity (Arif et al., 2020; Fadiji and Babalola, 2020; Zhang J. et al., 2021), developing tools to characterize them as accurately as possible is crucial.

Presently, the most widely used technique to perform such characterization resorts to metabarcoding, also known as amplicon High-Throughput Sequencing (HTS), which studies the microbial composition of bulk samples at an affordable cost. It is used in a wide range of laboratories and many sequencing and bioinformatics tools are available to generate and process sequencing data. The first step of metabarcoding involves amplifying a taxonomically informative region of microbial genomes. For archaea and bacteria, one or several hypervariable region(s) of the 16S rRNA-coding gene are often amplified (Pollock et al., 2018), whereas for fungi, a portion of the nuclear ribosomal Internal Transcribed Spacer (ITS) region, the 18S or the 28S rRNA-coding genes are targeted (Tedersoo et al., 2022). In a second step, the generated amplicons are sequenced, after which millions of sequencing reads are processed through a bioinformatics pipeline to infer the taxonomic composition of the sample and evaluate various diversity metrics. This metabarcoding approach enables fast and affordable assessments of species composition at an unprecedented scale (Taberlet et al., 2012).

In the case of endophytic microbiome metabarcoding, plant tissues are usually ground to extract total DNA and carry out subsequent amplifications. In addition to microbial DNA, most of the extracted genetic material comes from the plant itself, which can significantly hinder and often prevent any microbiome metabarcoding analysis. Indeed, the chloroplast 16S rRNA gene and the mitochondrial 18S rRNA gene display strong homologies with the bacterial 16S rRNA gene, whereas the plant ITS, 18S and 28S rRNA gene regions also show very similar patterns to their fungal counterparts. Owing to these homologies, the amplification of microbial loci is also accompanied by potentially significant co-amplification of plant DNA, especially when the extract contains a high proportion of plant DNA. Most of the time, this interfering co-amplification leads to sequencing results that are saturated in plant reads, with only a very small fraction belonging to microbial sequences. Authors facing this situation often have to disregard these samples (Morales Moreira et al., 2021; Azarbad et al., 2022) or use sequencing depths that are too low to accurately study sample microbial diversity (Žiarovská et al., 2020; Solanki et al., 2021).

One possible way to circumvent this problem is to increase the sequencing depth and subsequently remove plant sequences during bioinformatics processing of sequencing data. However, this strategy entails a significantly higher cost and does not guarantee that enough microbial reads will be recovered to achieve a reasonable sequencing depth. In practice, even deep sequencing (e.g., on HiSeq platforms) often fails to overcome the overwhelming presence of host DNA. Furthermore, the microbial diversity recovered may still be underestimated and/or biased, as plant DNA is still strongly amplified.

Other strategies rely on host DNA depletion, such as selective lysis of plant cells, methylation-based DNA digestion, or hybridization-based capture of microbial DNA. However, these approaches generally require additional laboratory steps, specialized reagents, higher costs, and their efficiency varies considerably across plant matrices. Another, simpler approach relies on a PCR-clamping principle, where modified nucleic acid oligomers are designed to hybridize to non-target DNA specifically (i.e., plant DNA in the present case) and block its amplification during the PCR process. This strategy directly reduces host co-amplification at the molecular level while remaining compatible with standard metabarcoding workflows. These oligomers can be either blocking primers or Peptide Nucleic Acids (PNAs). Blocking primers are oligonucleotides identical to traditional primers except that they display a C3-spacer at their 3′ end (Vestheim and Jarman, 2008). This spacer is a three-carbon chain (propyl group, -CH₂CH₂CH₂-) that acts as a structural block that prevents the formation of phosphodiester bonds between adjacent nucleotides, effectively halting the extension of the oligonucleotide chain. PNAs are another clamping solution. They are nucleic acid analogs in which the sugar-phosphate backbone is replaced by a peptide-like backbone. Despite this change, PNAs can still bind to complementary DNA or RNA sequences with high specificity and affinity (Karkare and Bhatnagar, 2006). Their uncharged character means that they can form PNA-DNA duplexes that are stronger than DNA–DNA duplexes. Regardless of the type of clamp, they are always used in combination with traditional PCR primers. The clamp (blocking primer or PNA) is designed to hybridize somewhere within the region amplified by traditional primers, at a site that may or may not overlap with the hybridization of one of the traditional PCR primers. The standard configuration uses two PCR primers with one clamp, but more than one clamp can be used in the same PCR if needed. PNAs were demonstrated to be more efficient at blocking non-target DNA than blocking primers (Lefèvre et al., 2020). They are, however, more expensive, whereas blocking primers could represent a more affordable compromise in preventing unwanted DNA from being amplified. PNAs described as universal and designed to block the amplification of chloroplast and mitochondrial DNA already exist (Lundberg et al., 2013). However, these PNAs display mismatches for various plant lineages, and even a single mismatch can significantly increase the level of contamination by plant DNA in sequencing results (Fitzpatrick et al., 2018). In such a situation, the best option is to design a new PNA, more suitable for the case at hand (Víquez-R et al., 2020).

Researchers generally design only one PNA to provide an answer to a given case study (Kawasaki and Ryan, 2021). It is also possible to design two PNAs that target the same locus while simultaneously blocking chloroplast and mitochondrial DNA amplifications in the same PCR (Lefèvre et al., 2020). Even though such a blocking system is efficient, it still targets only one locus, which is a considerable drawback when attempting to determine relevant estimates of both bacterial and fungal diversities. Indeed, it is now well established that sample composition should be assessed with more than one metabarcoding marker to avoid possible biases from taxa under−/over-amplification and database gaps, among others (Arulandhu et al., 2017; Adamowicz et al., 2019; Corse et al., 2019). Different regions of the 16S and ITS loci capture complementary taxonomic signals and resolution levels across microbial lineages, which can strongly influence community structure inference.

Consequently, this work was designed to develop new blocking systems that can be used to study the wheat-associated endophytic microbiome. Considering its worldwide importance in both the food and feed agro-industries, bread wheat (*Triticum aestivum ssp. aestivum*) was chosen as the host model. Both PNA and blocking primer options were explored to develop four blocking systems in total, i.e., two dedicated to the V4 and V5V7 regions of the bacterial 16S rRNA gene, and two dedicated to the ITS1 and ITS2 regions of fungal DNA. Additionally, balanced and unbalanced bacterial and fungal mock communities were also developed to demonstrate the absence of off-target inhibition during the amplification of microbial sequences.

## 2 Materials and methods

### 2.1 Plant material and DNA extraction

Several hundred wheat leaves from the Cubitus cultivar were collected from a conventional farming field in October 2022 in Peruwelz (Wallonia, Belgium) before being pooled and stored at −20 °C. These leaves were harvested at the BBCH 28 stage from a field where wheat was planted as a winter cover crop. The sampling locations were selected to ensure a homogeneous distribution across the field while avoiding a small, sloped area. To remove epiphytic communities, the surface of the leaves was sterilized in successive baths of 70% ethanol for 60 s, 1.5% sodium hypochlorite for 10 min and rinsed five times with sterile ultrapure water. The plant material was then ground into a fine powder in liquid nitrogen using sterile mortar and pestle. The DNeasy PowerSoil Pro kit (QIAGEN) was used to extract total DNA from 150 mg of leaf powder following the manufacturer’s instructions. The isolated DNA was eluted in 100 μL and quantified using a Qubit 4 fluorometer (ThermoFisher Scientific). DNA quality was confirmed using a Nanodrop One spectrophotometer (ThermoFisher Scientific) and only samples whose absorbance ratios were within the expected ranges (A260/A280: 1.8–2.0; A260/A230: above 2.0 and ideally 2.0–2.2) were retained. Negative controls were included at both the DNA extraction and PCR stages. They were systematically checked by agarose gel electrophoresis and by fluorometric (Qubit) and spectrophotometric (Nanodrop) quantification to confirm the absence of detectable contamination.

### 2.2 Development of mock communities

#### 2.2.1 Bacterial mock communities

To verify that the developed clamps did not inhibit bacterial amplification, a first mock community was created by mixing in known proportions DNA from 17 bacterial species commonly found in association with small grain cereals: *Bacillus subtilis*, *Burkholderia anthina*, *Enterobacter tabaci*, *Glutamicibacter creatinolyticus*, *Methylobacterium bullatum*, *Microbacterium oxydans*, *Pantoea agglomerans*, *Pedobacter foliorum*, *Pseudomonas cichorii*, *P. fuscovaginae*, *P. lurida*, *P. sivasensis*, *P. syringae*, *Sphingobacterium thalpophilum*, *Sphingomonas albertensis*, *Staphylococcus equorum* and *Xanthomonas translucens* (Braun-Kiewnick et al., 2025; Chen et al., 2022; Delitte et al., 2024). Additional information about this mock community is provided in Supplementary material 1. The DNA was extracted from individual liquid cultures using the DNeasy PowerSoil Pro Kit (QIAGEN) and its concentration was measured using a Qubit 4 fluorometer (ThermoFisher Scientific). Based on the concentration of each individual DNA extract, the length of each bacterial genome and the estimated 16S copy number per bacterial genome, a normalization factor was calculated to obtain a final DNA mix with 16S gene copy numbers from each species as close as possible to each other. Details on the calculation of normalization factors are provided in Supplementary material 1. For the last normalization step, qPCR amplifications were carried out with the primer sets 341F (5’-CCTACGGGAGGCAGCAG-3′)/534R (5’-ATTACCGCGGCT GCTGGCA-3′) and Eub338 (5’-ACTCCTACGGGAGGCAGCAG-3′)/Eub518 (5’-ATTACCGCGGCTGCTGG-3′) targeting the 16S rRNA gene as follows: initial denaturation at 95 °C for 2 min, followed by 40 cycles at 95 °C for 15 s, 53 °C for 30 s and 72 °C for 1 min. This assembled mock community, designed to contain the same expected number of 16S gene copies for each bacterial species in the mix, was named the ‘balanced bacterial mock community’. In addition, a second bacterial mock community with an unbalanced species profile was developed and named the ‘unbalanced bacterial mock community’. The expected species proportions in both mock communities are provided in Supplementary material 1.

#### 2.2.2 Fungal mock communities

Two fungal mock communities were developed, featuring either a balanced or an unbalanced profile of fungal species in the mixture. The 14 species used were selected on the basis of their known associations with wheat, other cereals or soil (*Zymoseptoria tritici*, *Microdochium* spp., *Fusarium graminearum*, *F. poae*, *Ramularia collo-cygni*, *Rhizoctonia solani*, *Colletotrichum coccodes*, *Globisporangium ultimum*, *Oculimacula yallundae*, *Epicoccum nigrum*; Dean et al., 2012; Moya-Elizondo et al., 2015; Nielsen et al., 2011; Zhang X. et al., 2021) or their application in the biocontrol of wheat-associated pathogens (*Trichoderma viride*, *Chaetomium globosum*, *Trichothecium roseum*; John et al., 2010; Zhu et al., 2022; Feng et al., 2023). Additionally, one exogenous species, *Phyllosticta citricarpa*, was included to check for the absence of contamination. Detailed information on the composition of these fungal mock communities and the DNA extraction methods used is provided in Supplementary material 2.

### 2.3 Blocking systems to amplify bacterial DNA

**Figure 1:**
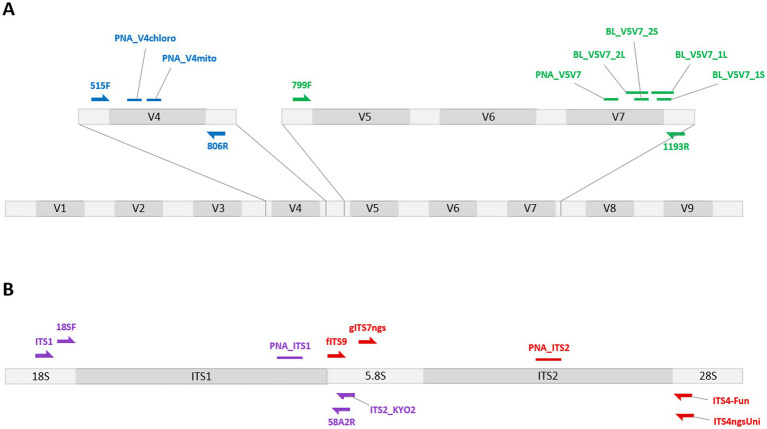
Localization of the different PCR clamps developed in this work, together with the traditional primers used. The PCR clamps were developed to block the amplification of wheat sequence counterparts to marker regions traditionally targeted when studying bacterial **(A)** or fungal **(B)** communities. The blocking systems targeting the V5V7 region (green) allowed the efficiency of blocking primers (BL_*) to be compared with that of a peptide nucleic acid (PNA). The PNA option was retained for the subsequent development of other blocking systems targeting the chloroplast 16S or the mitochondrial 18S V4 region (blue), and the ITS1 (purple) and ITS2 (red) sub-regions. The developed PCR clamps are represented by small horizontal bars, and the traditional primers used by semi-arrows.

Different blocking systems have been designed to target either the V5V7 or the V4 region of the 16S rRNA gene (Figure 1A). To design PCR clamps, 16S rRNA chloroplast sequences and 18S rRNA mitochondrial sequences from bread wheat were retrieved from NCBI and aligned using Geneious Prime (v2022.2.1) with 16S rRNA gene sequences from a wide set of bacterial species that are generally found in the wheat endophytic microbiome (Supplementary material 3). This sequence alignment facilitated the identification of conserved and variable regions, enabling the selection of clamp hybridization sites that are strictly conserved in wheat and highly divergent among bacterial species.

On its own, the primer set selected to amplify the V5V7 region of the bacterial 16S rRNA (799F/1193R) is sufficient to prevent the amplification of wheat chloroplast DNA because of the presence of several SNPs between the primers and their putative hybridization site in chloroplast sequences (Hanshew et al., 2013). However, this primer set still amplifies wheat mitochondrial sequences. Both blocking primers (carrying a C3-spacer at their 3′ ends) and PNAs were developed to evaluate their respective efficiencies in blocking the co-amplification of plant DNA (Table 1). All these clamps, except for BL_V5V7_1S, take advantage of the ~320 bp insertion present in this region of the wheat 18S rRNA gene. For the blocking primers, to determine whether a greater difference between the annealing temperatures of the blocking and traditional primers would increase the clamp efficiency, the blocking oligomers were short (*S primers) or long (*L primers) in design. BL_V5V7_1S and BL_ V5V7_1L were designed to hybridize to sites other than those of BL_ V5V7_2S and BL_ V5V7_2L. This allowed us to test whether the competitive approach (where universal PCR primers and blocking oligomers overlap) would be more effective than the elongation arrest approach (where the clamp hybridizes between the forward and reverse primers). To develop the PNA clamp (PNA_V5V7), we followed these guidelines, which can be used by researchers aiming to develop a new PNA clamp:

- A From NCBI, download reference sequences from the host and a broad range of microorganisms known to be associated with it, and align them.
- B Use the formulas provided in the cells of the Supplementary material 3 Excel datasheets to split aligned sequences and easily identify conserved and variable nucleotides at each position.
- C Identify potential clamp hybridization sites that are strictly conserved in host sequences but are highly divergent among microbial sequences.
- D Use the PNA tool^1^ to ensure that the candidate hybridization sites match the following criteria:

- (i) an annealing temperature higher than that of the PCR primers,
- (ii) a melting temperature (Tm) above the extension temperature,
- (iii) a length between 12 and 21 bases,
- (iv) no self-complementary stretches, and
- (v) a purine content lower than 50%, avoiding purine stretches of more than four residues.

E Use the Excel formulas in Supplementary material 3 to calculate the number of mismatches between each candidate PNA and the microbial sequences included in the alignment.
F Select the candidate clamp exhibiting the highest number of mismatches to microbial sequences.

**Table 1:** Nucleotide sequences of traditional primers and PCR clamps developed in this study.

Targeted region	Oligonucleotide name	Oligonucleotide sequence (5′- > 3′)	Oligonucleotide type	Reference
16S rRNA gene V5V7 (Bacteria)	799F (f)	AACMGGATTAGATACCCKG	Traditional primers	Chelius and Triplett (2001)
	1193R (r)	ACGTCATCCCCACCTTCC		Bodenhausen et al. (2013)
	BL_V5V7_1S	GGACTGCCAGTGAGATACTGGAG	Blocking primers	This study
	BL_V5V7_2S	AAAGGTGCGTGCCGCA		
	BL_V5V7_1L	CTCACGAGGGACTGCCAGTGAGATACTGGAGGAAGG		
	BL_V5V7_2L	CGCTCCGAAACAAAGAAAAAGGTGCGTGCCGCA		
	PNA_V5V7	CCCACGGAGACCTACCT	PNA	
16S rRNA gene V4 (Bacteria)	515F (f)	GTGCCAGCMGCCGCGGTAA	Traditional primers	Caporaso et al. (2011)
	806R (r)	GGACTACHVGGGTWTCTAAT		
	PNA_V4chloro	GCGTCTGTAGGTGGCTTTTC	PNA	This study
	PNA_V4mito	CGGAATGCTCTCGAAACC		
ITS1 (Fungi)	ITS1 (f)	TCCGTAGGTGAACCTGCGG	Traditional primers	White et al. (1990)
	58A2R (r)	CTGCGTTCTTCATCGAT		Martin and Rygiewicz (2005)
	18SF (f)	GTAAAAGTCGTAACAAGGTTTC		Findley et al. (2013)
	ITS2_KYO2 (r)	TTYRCTRCGTTCTTCATC		Toju et al. (2012)
	PNA_ITS1	CTATTTAATCCACACGACTCTCGG	PNA	This study
ITS2 (Fungi)	gITS7ngs (f)	GTGARTCATCRARTYTTTG	Traditional primers	Tedersoo and Lindahl (2016)
	ITS4ngsUni (r)	CCTSCSCTTANTDATATGC		
	fITS9 (f)	GAACACAGCGAAATGTGA		Ihrmark et al. (2012)
	ITS4-Fun (r)	AGCCTCCGCTTATTGATATGCTTAART		Taylor et al. (2016)
	PNA_ITS2	CGGCATCTGGTCCCTCGTCTC	PNA	This study

(f), forward primer; (r), reverse primer. Universal Illumina adaptors were attached at the 5′ ends of the forward and reverse primers (ACACTCTTTCCCTACACGACGCTCTTCCGATCT and GACTGGAGTTCAGACGTGTGCTCTTCCGATCT, respectively).

To amplify the V4 region, the primers 515F and 806R were chosen as they are among the most commonly used primers in microbiome studies, such as the Earth Microbiome Project (Gilbert et al., 2014). This primer set can co-amplify both chloroplast and mitochondrial plant DNA. Given the superior results obtained with the PNA compared with the blocking primers in the V5V7 clamping assay, along with its greater ease of development, it was decided to retain the PNA as the most viable option for further designs. Two PNAs were therefore developed to block the amplification of either 16S rRNA chloroplast sequences (PNA_V4chloro) or 18S rRNA mitochondrial sequences (PNA_V4mito; Table 1). In both cases, clamps were designed to include at least five SNPs with the corresponding bacterial sequences, to avoid inhibiting the amplification of bacterial sequences.

### 2.4 Blocking systems to amplify fungal DNA

Similar to the approach for designing V5V7 and V4 blocking systems, PNA clamps were developed to target the ITS region (Figure 1B). To do this, wheat ITS reference sequences were aligned with those of fungal taxa often found in the wheat endophytic microbiome (Supplementary material 3). The wheat sequences were selected to cover a wide geographical distribution (Bulgaria, China, Germany, Iran, the Netherlands, and the United States). Among the selected fungal reference sequences, 40 belonged to the Ascomycota phylum (representing five classes) and 23 belonged to the Basidiomycota phylum (representing 8 classes). This sequence alignment facilitated the design of two PNA clamps to target the ITS1 and ITS2 sub-regions, respectively (Table 1). The PCR primers used to test the efficiency of these clamps, reported in Table 1, were selected for three main reasons: (i) they were previously shown to significantly co-amplify wheat DNA (Morales Moreira et al., 2021), (ii) they are recommended for fungal metabarcoding studies (Nilsson et al., 2019; Tedersoo et al., 2022), or (iii) they offer different taxonomic coverages (Nilsson et al., 2019).

### 2.5 PCR amplifications and high-throughput sequencing

A total of 18 PCR systems were tested, corresponding to eight bacterial and eight fungal amplification setups (see Sections 2.3 and 2.4 for primer and blocking agent details). For each PCR system (i.e., each primer set, with or without a blocking agent), nine identical PCR reactions were performed. These nine replicates were then subdivided into three groups of three reactions. Within each group, the three PCR products were pooled during the purification step to obtain one combined sample, resulting in three sequencing libraries per PCR system. In total, 54 sequencing libraries (18 PCR systems × 3 libraries per system) were generated and analyzed. Pooling three technical replicates at the purification stage was intended to minimize variability arising from individual PCR reactions while maintaining three independent sequencing replicates per system. This design provided a robust estimate of within-system reproducibility, while keeping technical variation under control.

All PCRs were carried out using 5 μL of 5X GoTaq® Flexi Buffer (Promega, Madison, WI, United States), 2.5 μL of 2 mM dNTP mix (ThermoFisher Scientific, Foster City, CA, United States), 1.5 μL of 25 mM MgCl_2_ (Promega, Madison, WI, United States), 1 μL of 10 μM forward and reverse primers (Eurofins Genomics, Köln, Germany, see Table 1) appended with Illumina universal adapters, 0.15 μL of GoTaq® G2 Flexi DNA Polymerase (Promega, Madison, WI, USA), 1 μL of DNA and nuclease-free water (QIAGEN, Hilden, Germany), resulting in a final volume of 23 μL. All reactions were supplemented with either 2 μL of nuclease-free water (no blocking condition) or 2 μL of one of the designed clamps (Eurogentec, Seraing, Belgium). The initial concentrations of the blocking primers and PNA were 50 μM and 25 μM, respectively. While developing the clamp, it was observed that diluting the starting DNA improved the blocking efficiency in cases where the clamp did not fully inhibit plant sequence amplification. This effect was consistent across several tests, indicating that high template concentrations may reduce the relative efficiency of the clamp. Therefore, DNA dilution was performed as an optimization step to enhance clamping performance. As primer pairs differed in their amplification efficiency, different DNA dilutions were applied to obtain bands of moderate intensity on agarose gel (sufficient for sequencing but not excessively strong). The DNA concentration used for each primer set is reported in Supplementary material 4.

Detailed information regarding thermal cycling and oligonucleotide combination for each clamping assay is provided in Supplementary material 5. For traditional and blocking primers, the annealing temperature was computed using the NEB Tm calculator.^2^ For PNAs, the melting temperature was computed using the PNA Bio design tool.^3^ The PNAs were designed to have a Tm far enough from the elongation temperature, i.e., as close to 80 °C as possible. Note that a PNA/DNA duplex will display a higher Tm than the corresponding DNA/DNA duplex.

Among the three sets of three identical PCR products, triplicate amplicons were pooled during purification using the NucleoSpin Gel and PCR Clean-up kit (Macherey-Nagel, Düren, Germany) to obtain three sequencing libraries per sample. The amplicon quality was verified by running 5 μL of PCR products on a 1.2% agarose gel and using a Nanodrop One spectrophotometer (ThermoFisher Scientific). Purified amplicons were quantified using a Qubit 4 fluorometer (ThermoFisher Scientific). The remaining 25 μL of purified amplicons were then sent to Eurofins Genomics (Köln, Germany) for the second PCR – allowing the addition of indexed sequencing adaptors for multiplexing – and high-throughput sequencing on an Illumina MiSeq device with the 2×300 bp chemistry. All sequences generated in this study are available in the NCBI sequence read archive under the BioProject number PRJNA1039717.

### 2.6 Bioinformatics analysis

The raw sequencing data were imported into QIIME2 for bioinformatics processing. Demultiplexed paired-end reads were denoised with DADA2 (Callahan et al., 2016) to generate amplicon sequence variants (ASVs). For this denoising, the length of the target-specific primers (i.e., without overhangs) was used to trim the 5′ ends of the reads. Quality plots were used to truncate the 3′ ends of the reads, ideally at the first position where the average phred score dropped below 30. For the other parameters, default settings implemented in the DADA2 QIIME2 plugin were used. Taxonomy was assigned to the ASVs using the q2-feature-classifier (Bokulich et al., 2018) classify-consensus-blast taxonomy classifier against the different reference databases. The SILVA 138 SSU database was used for 16S rRNA sequencing data. For fungal annotations, all ITS sequences from plants and fungi were retrieved from NCBI on April 4^th^ 2023 and were processed into a curated reference database using the DB4Q2 pipeline (Dubois et al., 2022). The resulting reference database is available at the following public repository: https://doi.org/10.6084/m9.figshare.26976538. In the specific case of V5V7 amplifications, very different amplicon lengths were observed between the plant and bacterial PCR products (740 bp vs. 415 bp, respectively). Since all the plant sequences were lost during read merging due to the excessive length of amplicons (it was not possible to merge forward and reverse reads due to the absence of an overlap), this loss represented a bioinformatic artifact rather than a true reduction of plant amplification. PCR clamping remained essential, as host DNA was still abundantly amplified during PCR, consuming reagents and sequencing depth, thereby reducing the number of microbial reads recovered and potentially biasing microbial community estimates. To ensure that the results reflected the actual level of co-amplification, the initial taxonomic analysis was carried out on pre-denoising sequencing data, while further taxonomic analyses (i.e., after the removal of plant reads) were performed on denoised data.

Statistical analyses were carried out using the q2-diversity plugin: the alpha and alpha-phylogenetic pipelines were used to compute alpha diversity metrics, and the significance of the observed differences was assessed via Kruskal-Wallis tests using the alpha-group-significance command.

## 3 Results

### 3.1 Assessment of blocking efficiency on field samples

The evaluation of clamp blocking efficiency was performed by applying them to the analysis of the endophytic microbiome of wheat field samples. In total, 9,351,822 raw reads were obtained across the 54 samples, with an average of 173,182 ± 47,996 reads per sample.

#### 3.1.1 16S rRNA gene blocking systems

**Figure 2:**
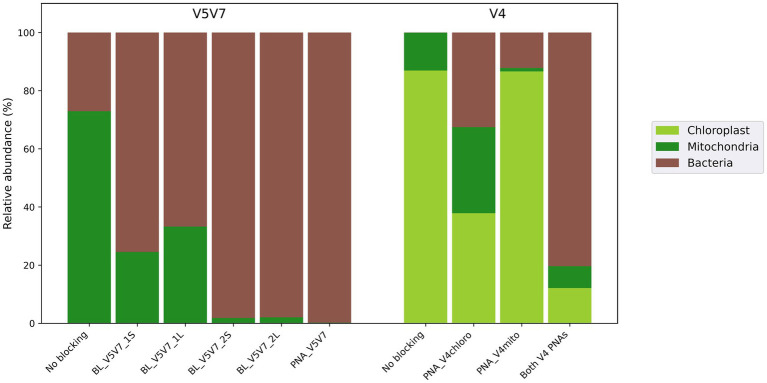
Efficiency of the clamps developed to block wheat DNA amplification when targeting bacterial communities. For the V5V7 assay, the clamps targeted only the 18S rRNA gene from wheat mitochondria since the primer set 799F/1193R naturally prevents the amplification of 16S rRNA sequences from wheat chloroplasts. The developed clamps were either blocking primers (BL_*) or a peptide nucleic acid (PNA). For the V4 assay, two PNAs were designed to block the amplification of wheat chloroplast and mitochondrial DNA, respectively.

The amplifications targeting the V5V7 region were carried out with the 799F/1193R primer set as it avoids amplifying chloroplast DNA on its own. Each developed PCR clamp was assessed for its efficiency to block mitochondrial DNA amplification by evaluating the amount of plant vs. bacterial amplicons and by studying the evolution of diversity metrics. Adding developed PCR clamps to the PCR mixture resulted in limited plant DNA amplification for all of them, with varying efficiencies (Figure 2). Under conditions without any blocking system, more than 70% of the sequencing reads originated from wheat. Importantly, because wheat amplicons were approximately 325 bp longer than bacterial ones (~740 bp vs. ~415 bp), Illumina paired-end sequencing tends to favor shorter inserts, thereby underestimating the true extent of host co-amplification. This interpretation is consistent with agarose gel profiles (data not shown), where the wheat band was much more intense than the bacterial band, indicating a stronger host amplification than suggested by the sequencing data. Consequently, in contexts where host and microbial amplicons are of comparable length, such as in most other primer–template combinations, the bias would not occur, and the impact of host DNA co-amplification on sequencing results would likely be even more pronounced.

Despite the fact that all blocking primers could (partially) inhibit wheat DNA amplification, oligonucleotides hybridizing in the middle of the amplicon (BL_V5V7_2S and *_2L – elongation arrest approach) presented a better bacterial-to-plant sequence ratio than those competing for the hybridization site with the reverse 1193R primer (BL_V5V7_1S and *_1L – competitive approach; Figure 2). In contrast, the short (*S primers) or long (*L primers) design of the clamp did not significant differ in this regard. For the PNA-based approach, in addition to the fact that the PCR conditions were more easily optimized, PNA_V5V7 achieved the best performance with almost 100% bacterial reads, statistically supported when compared to the blocking primer systems (*p* = 0.049).

When the V4 region of the bacterial 16S rRNA gene is targeted, the commonly used 515F/806R primers may co-amplify both the chloroplast 16S rRNA gene and the mitochondrial 18S rRNA gene. Therefore, two different PCR clamps had to be developed to block both chloroplast (PNA_V4chloro) and mitochondrial (PNA_V4mito) DNA amplification. The results demonstrated the benefit of developing these systems as, in the absence of clamps, only DNA from chloroplasts and mitochondria was amplified (Figure 2). Using only one of the two PNAs inhibited the amplification of the targeted sequences, but a significant number of plant sequences from the other organelle type remained. The results revealed that using both PNAs simultaneously was the most effective strategy, allowing for the recovery of more than 80% of the bacterial reads.

For both the V5V7 and V4 clamping assays, the number of represented genera and additional diversity metrics computed after the removal of plant sequences underlined the usefulness of these blocking systems (Table 2; Supplementary material 6). While the amplification of bacterial sequences without a clamp was almost completely inhibited for the V4 region, V5V7 amplifications resulted in the recovery of bacterial reads with 25 genera detected even without blocking systems (although at (very) low frequency). In both cases, the use of designed clamps led to a significant increase in the number of identified genera, and an overall increase in the diversity metrics. The only exception is for V5V7 amplifications without a blocking agent, where richness metrics (observed features and Faith PD) showed higher diversity values. Interestingly, while the analysis of plant and bacterial sequences shown in Figure 2 indicated better performance for the blocking primers using the elongation arrest approach (BL_V5V7_2* primers) than for the competitive approach (BL_V5V7_1* primers), this trend was not supported by diversity metrics, as a similar number of bacterial genera were recovered with both approaches (Table 2).

**Table 2:** Metrics reflecting the effectiveness of the developed PCR clamps.

A	Targeted region	Primer set	Blocking condition	Plant sequences^1^	Bacterial sequences^1^	Bacterial genera^2^
	16S rRNA gene V5V7 (Bacteria)	799F/1193R	No blocking	5 (c) + 32,754 (m)	12,163	25
			BL_V5V7_1S	7 (c) + 16,830 (m)	51,824	43
			BL_V5V7_1L	5 (c) + 29,075 (m)	58,373	37
			BL_V5V7_2S	9 (c) + 1956 (m)	107,240	43
			BL_V5V7_2L	5 (c) + 1988 (m)	96,726	40
			PNA_V5V7	2 (c) + 229 (m)	104,078	44
			*p-value*	*0.009*	*0.029*	*0.053*
	16S rRNA gene V4 (Bacteria)	515F/806R	No blocking	58,505 (c) + 8,749 (m)	48	4
			PNA_V4chloro	28,572 (c) + 22,378 (m)	24,567	37
			PNA_V4mito	68,645 (c) + 941 (m)	9,652	27
			PNA_V4chloro + PNA_V4mito	7,141 (c) + 4,450 (m)	47,404	51
			*p-value*	*0.043*	*0.016*	*0.023*
B	Targeted region	Primer set	Blocking condition	Plant sequences^1^	Fungal sequences^1^	Fungal genera^2^
	ITS1 (Fungi)	18SF/ITS2_KYO2	No blocking	34,684	5	0
			PNA_ITS1	13,796	26,269	24
			*p-value*	*0.050*	*0.046*	*0.046*
	ITS1 (Fungi)	ITS1/58A2R	No blocking	66,877	3,623	*9*
			PNA_ITS1	1738	32,752	*23*
			*p-value*	*0.050*	*0.050*	*0.077*
	ITS2 (Fungi)	gITS7ngs/ITS4ngsUni	No blocking	53,355	16	1
			PNA_ITS2	6,631	21,297	30
			*p-value*	*0.050*	*0.050*	*0.046*
	ITS2 (Fungi)	fITS9/ITS4-Fun	No blocking	51,278	1769	11
			PNA_ITS2	4,966	14,308	29
			*p-value*	*0.050*	*0.050*	*0.050*

These clamps were used to block the amplification of wheat DNA using primer sets targeting bacterial (A) or fungal (B) regions. For all samples, the metrics were measured on three replicate sequencing libraries and averaged to report the mean values in this table. The detailed results for each replicate are provided in Supplementary material 7. *p*-values refer to the results of Kruskal-Wallis group significance tests. ^1^Numbers refer to denoised reads except for the V5V7 assay where all plant sequences were lost during read merging given their significantly higher length. For this region, plant and bacterial sequences were therefore counted before denoising. ^2^Computed after removing plant reads. (c), chloroplast; (m), mitochondria.

With the V4 clamping assay, amplifications without any PNA led to a very low number of bacterial reads, corresponding to only four genera that were hardly detected. The use of both PNAs together had a synergistic effect, as it allowed the recovery of the highest bacterial diversity with 51 genera detected. This approach also resulted in the greatest number of bacterial reads by far, which should be an interesting feature when the aim is to detect rare taxa.

An analysis of the taxonomic compositions inferred with the V5V7 and V4 PCR systems revealed very concordant results (Supplementary material 8). Amplifications performed without a blocking agent failed to accurately characterize bacterial populations both qualitatively and quantitatively, resulting in significantly reduced diversity and artificial over-representation of certain taxa, such as *Pseudomonas* and *Pantoea*. In contrast to amplifications with clamps, this approach also generated a substantial proportion of unassigned reads. Interestingly, the results obtained with clamps showed similar trends in both regions. This provides a good indication that the developed clamps do not inhibit the amplification of bacterial sequences.

#### 3.1.2 ITS blocking systems

**Figure 3:**
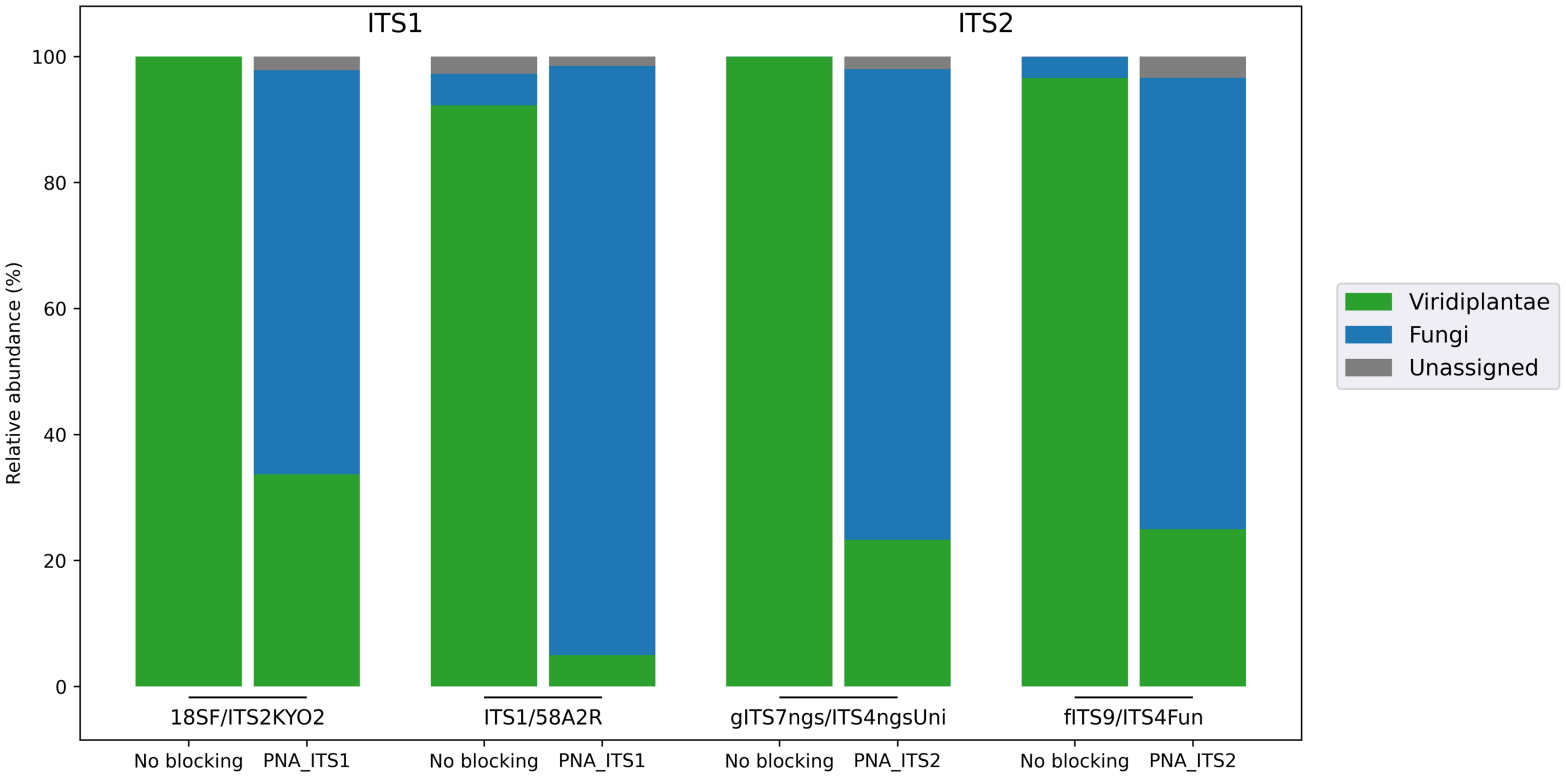
Efficiency of the clamps developed to block wheat DNA amplification when targeting fungal communities. Two PNAs were developed to target the first and second portions of the internal transcribed spacer (PNA_ITS1 and PNA_ITS2). To evaluate clamp efficiency, four primer sets were selected owing to their differences in taxonomic coverage.

Two PNA clamps targeting the ITS1 and ITS2 regions, respectively, of wheat sequences were developed to study fungal communities associated with this host. Their blocking efficiency was evaluated using four primer sets targeting one of these two regions (Table 1). Whereas amplifications carried out without a blocking agent resulted in (almost) exclusively plant reads, adding the developed PNAs substantially reduced the proportion of plant reads, to the benefit of fungal sequences (Figure 3). This provided a much better picture of the fungal communities studied, with significantly higher numbers of genera detected compared with amplifications without PNA, and better diversity metrics (Table 2; Supplementary material 6). The analysis of taxonomic composition after the removal of plant reads further highlighted the effectiveness of the developed clamps, with significantly more detected genera when using a PNA compared to when no PNA was used (Supplementary material 9). Interestingly, the fungal composition inferred from the different primer sets with PNA was similar overall, but it was less consistent than what was observed in the bacterial assay (V5V7 vs. V4). This is in line with the known heterogeneity of the results generated by most fungal primer pairs. The primers used in this work were actually selected to display different taxonomic coverages, some of them covering ~90% of fungi, others almost 100%, or even other eukaryotes (Nilsson et al., 2019).

### 3.2 Checking the absence of inhibition of microbial sequence amplification

**Figure 4:**
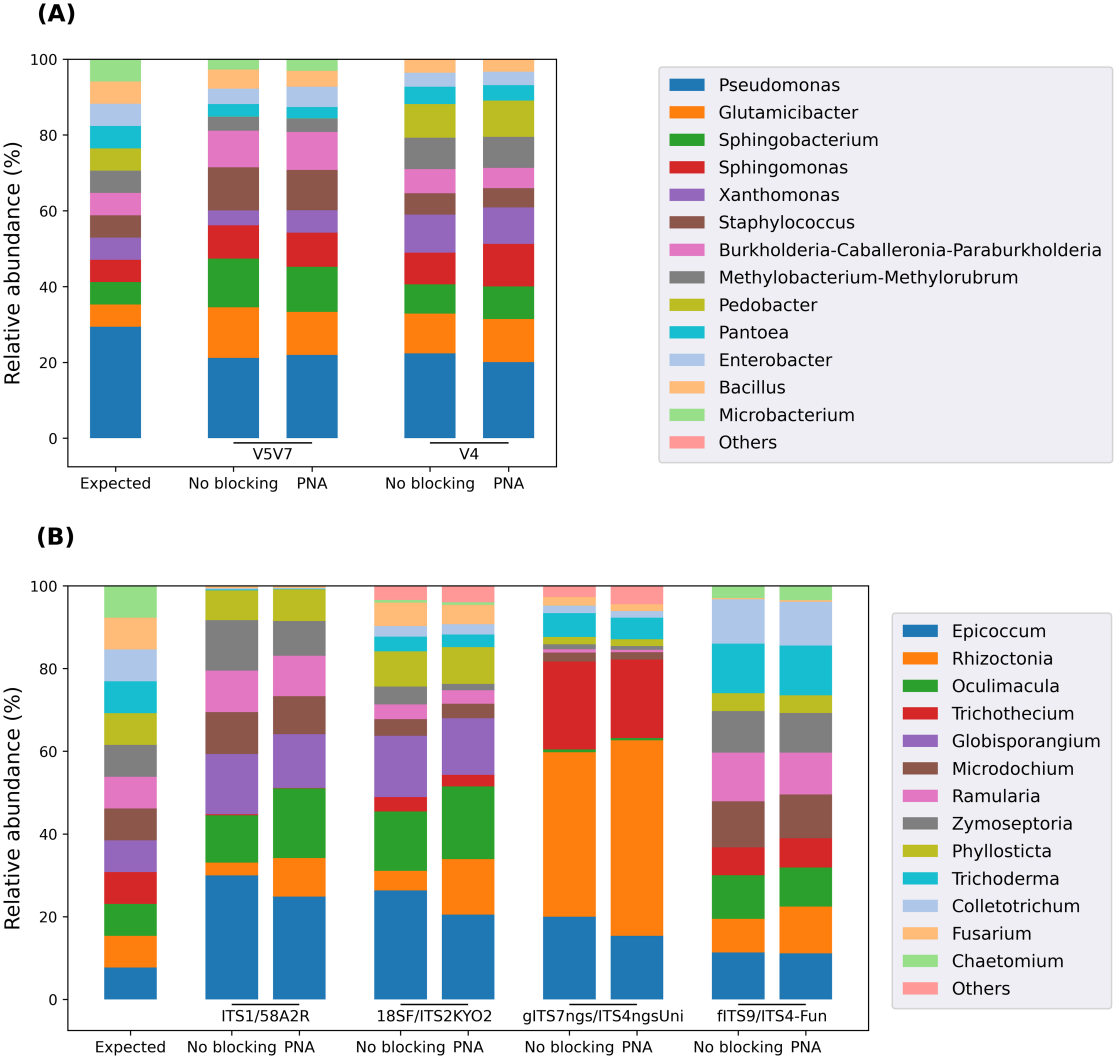
Taxonomic composition of the balanced bacterial **(A)** and fungal **(B)** mock communities assessed using a metabarcoding approach, with or without developed PCR clamps. The similarity of the results obtained with or without PNA for each primer pair and each mock community validated the absence of inhibition in microbial sequence amplification. Since the Illumina metabarcoding approach reliably resolves only to the genus level, the results for the five *Pseudomonas* species were merged in panel **A**.

To further confirm that the developed clamps did not interfere with the amplification of bacterial DNA, PCRs were also performed using balanced and unbalanced mock communities, with or without a blocking agent (Figure 4; Supplementary material 10). The results obtained using the balanced bacterial mock community showed a very good correlation between the expected and observed taxon abundances for both targeted regions (Figure 4A). Only *Pedobacter* and *Microbacterium* were not detected using the V5V7 and V4 regions, respectively. In both cases, deeper analysis revealed that these discrepancies were due to mismatches between the reverse PCR primers and their respective hybridization sites in the sequences from these genera (1 SNP between 806R and *Microbacterium* 16S sequence; 2 SNPs between 1193R and *Pedobacter* 16S sequence). Furthermore, the results were almost identical, regardless of whether a PNA was used. This confirmed that the blocking capacity of the developed PNAs was indeed targeted against the amplification of plant DNA, without any inhibitory effect on bacteria.

On the fungal side, the results confirmed that achieving taxa proportions close to the expected values was more challenging (Figure 4B). This can be explained by the difficulty in obtaining a mixture with the same number of ITS sequence copies for each fungal species due to the lack of genomic information. In addition, the taxonomic biases caused by certain primer sets, observed above with field samples, are also evident here. For example, *Colletotrichum* and *Trichoderma* were missed by the ITS1/58A2R primers, whereas *Globisporangium* was missed by both ITS2 primer sets, among others. Nevertheless, these results validated the intended purpose of this mock community: the introduction of PNAs into the reaction mixture did not cause any inhibitory effect on the amplification of fungal sequences.

Using unbalanced instead of balanced bacterial and fungal mock communities led to the same conclusion: the developed PNAs did not inhibit microbial sequence amplification, even for taxa present in low proportions (Supplementary material 10).

## 4 Discussion

### 4.1 Advances in wheat clamping strategies

This work represents a significant advance over previous studies for several reasons. To our knowledge, this is the first study to present a comprehensive set of validated clamps that enable the accurate characterization of both bacterial and fungal endophytic communities, providing a complete view of the microbial members within the pyllosphere endophytic microbiome of wheat. The study of bacterial and fungal communities from different perspectives is possible owing to the inclusion of at least two clamps for each microbial category (e.g., V4 and V5V7 for bacteria, ITS1 and ITS2 for fungi), thus ensuring more robust and reliable results. Moreover, the efficiency of the clamps was rigorously demonstrated using field samples and further validated by confirming the absence of off-target effects on microbial DNA amplification. This was achieved through experiments with four distinct mock communities and the alignment of a wide set of microbial reference sequences, a level of validation not reported in previous studies. Additionally, this article offers a comprehensive set of guidelines for researchers aiming to develop PNA clamps for other host organisms, with clear and detailed instructions that make the methodology accessible and reproducible.

### 4.2 Amplifications without a blocking system lead to strongly biased results

Since the starting material from field samples was mostly composed of wheat DNA, the results showed that amplifications performed without a clamp significantly underestimated microbial diversity. In contrast, all the developed clamps effectively eliminated or at least substantially mitigated this issue.

An additional, less common, phenomenon was observed in situations where PCRs without a blocking agent led to several thousand bacterial reads. The amplifications targeting the V5V7 region without a blocking system revealed a surprisingly high number of ASVs. Several trends can be extracted from the analysis of these ASVs. First, the ASVs belonging to the samples without a blocking agent presented a low frequency and unusual distributions. Indeed, most of the time, they were present in only one of the three replicates (only 30 out of the 475 ASVs detected in these samples were present in the three replicates). Second, ASVs observed in samples with clamps were detected to a much lesser extent (and sometimes even not at all) in samples without a blocking agent. Third, despite the high number of ASVs in samples without a clamp, fewer bacterial genera were noted than in other samples where a clamp was used. Finally, each of the five V5V7 clamps designed have their own design and therefore different hybridization sites. Given that the use of these clamps provided almost identical taxonomic results for each of them (see Supplementary material 8), it is very unlikely that the higher richness observed in samples without a blocking system is due to an off-target effect of the clamps toward bacterial sequences (since the scale of the bacterial inhibitory effect would have fluctuated depending on the clamps used). This is further supported by the fact that the taxonomic compositions inferred from samples with a blocking system are also very similar to those observed for the V4 region. Furthermore, each of the clamps was designed to have a very different sequence from those of bacterial reference sequences (at least 5 SNPs), which is another guarantee of the targeted action of the clamps, since a single mismatch destabilizes the PNA–DNA hybrids (Zhao et al., 2016). All these observations, in addition to the unequivocal results shown in Figure 4, lead us to advise against the use of amplicon HTS without a clamping system when the starting material is severely contaminated by host DNA. Indeed, there is a major risk of significantly underestimating microbial diversity and in some cases, it can also generate many low-frequency noisy reads (with a number of detected genera remaining low).

During denoising, it was observed that this step reduced the number of host reads because wheat amplicons were significantly longer, which prevented the merging of forward and reverse reads. It must be noted that this is a phenomenon specific to wheat sequences and does not mitigate the detrimental effects of host DNA co-amplification. Therefore, PCR clamping remains crucial even for the V5V7 region. Bioinformatic filtering can remove contaminant sequences *post hoc*, but it cannot recover the sequencing depth or diversity lost when plant DNA dominates amplification. By preventing this issue at the PCR stage, clamping ensures a more representative amplification of microbial DNA and a more reliable assessment of endophytic community composition.

### 4.3 Applicability of the developed clamps

The V5V7 assay underscored the relative ease of developing PNA clamps, provided that the guidelines mentioned in section 2.3 are followed. In comparison, blocking primers required more time to determine the best PCR conditions, and exhibited (slightly) reduced performance compared with those of PNA_V5V7. These reasons explain why PNAs were retained for the development of clamps targeting other regions. However, whether blocking primers would have performed worse than PNAs in other regions remains to be determined. Therefore, blocking primers remain a viable option to explore, for researchers seeking a solution at reduced cost. In practical terms, PNA clamps are substantially more expensive to purchase than blocking primers (≈ €575–830 for 50 nmol of PNA vs. €44–55 for 10 nmol of blocking primers, prices excluding tax). However, at the working concentrations used here, this corresponds to an additional cost of approximately €0.6–0.8 per PCR when adding a PNA, and about €0.5 per PCR when adding a blocking primer.

Considering the number of bacterial reads vs. plant reads, blocking primers using the elongation arrest strategy was more efficient than the competitive approach was, whereas the opposite was observed elsewhere (Von Wintzingerode et al., 2000; Vestheim and Jarman, 2008). In contrast, comparing the results provided by the blocking primers with a short (*S) or a long (*L) design revealed that a higher difference between the annealing temperatures of the blocking and traditional primers did not improve the blocking efficiency.

The bacterial clamps developed in this study were associated with the primer sets 515F/806R and 799F/1193R, which amplify the V4 and V5V7 regions of the 16S rRNA-coding gene, respectively. However, their applicability is not limited to these primers/regions. Many other primer sets are used in the literature to target (slightly) different 16S regions, such as the V3V4 (Sahu et al., 2021), V3V5 (Lasa et al., 2019), V4V5 (Yang et al., 2020) and V6V8 (Yurgel et al., 2018), for example. Since all these regions span the hybridization sites of the blocking agents developed in this work, these clamps can also be used in similar experimental designs. This observation is also valid for fungal clamping assays since a very wide set of primers are used in the literature to amplify the ITS1 and ITS2 sequences from fungi (Nilsson et al., 2019). Moreover, with the advent of third-generation HTS technologies, bacterial populations can be profiled by sequencing the full 16S rRNA gene at once (Johnson et al., 2019; Matsuo et al., 2021), or even the whole ribosomal operon 16S-ITS-23S (Kinoshita et al., 2021; Dubois et al., 2024; Lengrand et al., 2024), which significantly increases the taxonomic resolution of these approaches. Similarly, mycologists now take advantage of long-read technologies to sequence the full fungal operon 18S-ITS1-5.8S-ITS2-28S (D’Andreano et al., 2021; Lu et al., 2022; Ohta et al., 2023). The developed clamps can, of course, also be used in such cases since they are part of these larger fragments. Different clamps (e.g., PNA_ITS1 and PNA_ITS2) can even be used at the same time to increase efficiency if needed.

The plant material used in this work to check the efficiency of the developed clamps originated from one wheat cultivar and was collected at one location and one time point, which could raise the question of the wheat and microbial representativeness of these samples. The clamps were designed based on the alignment of different wheat sequences downloaded from the NCBI database; therefore, they are representative of more cultivars than just the one harvested in this study. In addition, the list of bacteria/fungi used to develop the clamps was extensive and spanned a very wide diversity of microorganisms. Using this list, we ensured that the clamps were very different from their closest microbial sequence (at least 5 SNPs), which prevents the clamps from hybridizing to these sequences. Some of the clamps anneal to a DNA insertion that does not even exist in bacterial sequences. Moreover, the different mock communities assembled in this work contained a wide diversity of microorganisms commonly associated with wheat, and the absence of off-target inhibition was demonstrated for all of them.

Interestingly, the sequences of the clamps developed in this work were found in a wide range of reference sequences from wheat relatives (Supplementary material 11). This homology was highlighted in tetraploid and hexaploid species from the *Triticum* genus and in *Aegilops speltoides*, *Ae. tauschii* and *Triticum urartu*. These are diploid species considered to be the ancestors of wheat and therefore exhibit strong genetic similarities with wheat (Dubois et al., 2016). The hybridization sites of the clamps could also be identified in the sequences of other cereal species of the Poaceae family (barley, rye and maize), although some mismatches were found for rye. Taken together, these observations indicate that the designed clamps should have a broader range of applicability, beyond wheat, to effectively block host DNA amplification.

### 4.4 Placing this study in the context of previous works

Several PNA clamps were previously developed to block the amplification of plant DNA. One of the most notable studies was conducted by Lundberg et al. (2013), who designed ‘universal’ PNA clamps to block the amplification of plastid (pPNA) and mitochondrial (mPNA) plant sequences. Although the match between these PNAs and a list of plant sequences (without wheat) had been theoretically verified, their efficiency was precisely characterized for only two species. Even with *Oryza sativa*, a species displaying perfect matches with the PNAs, the authors struggled to effectively block the amplification of host plastid and mitochondrial DNA. These considerations and the lack of coverage toward Asteraceae members prompted Fitzpatrick et al. (2018) to develop a modified pPNA for this taxonomic family. Although the modified PNA successfully reduced host contamination in Asteraceae species, it increased host DNA co-amplification in non-Asteraceae species. All these observations led to the conclusion that universal PNAs are unfortunately not effective for a large number of plant species (Alibrandi et al., 2020), highlighting the need to develop case-specific clamps to ensure reliable results.

A final, less common phenomenon may obstruct the use of ‘universal’ PNAs: the transfer of plastid or mitochondrial DNA fragments to the nuclear genome, which has an important role in the evolution of eukaryote genomes (Rousseau-Gueutin et al., 2012; Yoshida et al., 2014). Most of the time, these nuclear integrants display sequence divergence compared with the original organelle sequence. This prevents the use of ‘universal’ PNA clamps in such cases, since the amplification of plastid/mitochondrial DNA may be blocked, but not that of the integrant (recently observed in our laboratory).

Consequently, we are strongly convinced that the best option is to develop PNA clamp(s) specifically designed for the host under study. In this work, tools were developed for wheat as a host, and the methodology was described in detail to ensure easy adaptation to any other host.

## Data Availability

The raw sequencing data generated in this work is available at the NCBI Sequence Read Archive under the BioProject number PRJNA1039717. The reference database developed in this study, dedicated to ITS sequences from fungi and plants, is available in the following public repository: https://doi.org/10.6084/m9.figshare.26976538.

## Footnotes

^1^ https://www.pnabio.com/support/PNA_Tool.htm

^2^ https://tmcalculator.neb.com/

^3^ https://www.pnabio.com/support/PNA_Tool.htm
