# Supplementary material for: Improving the profiling of wheat bacterial and fungal endophytic communities—a PCR clamping approach
Source: Front Microbiol. 2025 Oct 31;16:1690976. doi: 10.3389/fmicb.2025.1690976 (PMC12615186; doi:10.3389/fmicb.2025.1690976)
Supplement: Supplementary file 8 [file Data_Sheet_1.PDF]

<u>Supplementary material 2. Detailed information regarding the species included in the balanced and unbalanced fungal mock communities.</u>

1. Taxonomic lineages of fungal species selected to assemble mock communities

**Kingdom**	**Phylum**	**Class**	**Order**	**Family**	**Genus**	**Species**
Fungi	Ascomycota	Sordariomycetes	Sordariales	Chaetomiaceae	*Chaetomium*	*globosum*
Fungi	Ascomycota	Sordariomycetes	Glomerellales	Glomerellaceae	*Colletotrichum*	*coccodes*
Fungi	Ascomycota	Dothideomycetes	Pleosporales	Didymellaceae	*Epicoccum*	*nigrum*
Fungi	Ascomycota	Sordariomycetes	Hyporceales	Nectriaceae	*Fusarium*	*graminearum*
Fungi	Ascomycota	Sordariomycetes	Hyporceales	Nectriaceae	*Fusarium*	*poae*
Fungi	Ascomycota	Sordariomycetes	Xylariales	Microdochiaceae	*Microdochium*	*spp.*
Fungi	Ascomycota	Leotiomycetes	Helotiales	Ploettnerulaceae	*Oculimacula*	*yallundae*
Fungi	Ascomycota	Dothideomycetes	Botryosphaeriales	Phyllostictaceae	*Phyllosticta*	*citricarpa*
Fungi	Oomycota	Oomycetes	Pythiales	Pythiaceae	*Globisporangium*	*ultimum*
Fungi	Ascomycota	Dothideomycetes	Mycosphaerellales	Mycosphaerellaceae	*Ramularia*	*collo-cygni*
Fungi	Basidiomycota	Agaricomycetes	Cantharellales	Ceratobasidiaceae	*Rhizoctonia*	*solani*
Fungi	Ascomycota	Sordariomycetes	Hyporceales	Hypocreaceae	*Trichoderma*	*viride*
Fungi	Ascomycota	Sordariomycetes	Hyporceales	Hypocreales incertae sedis	*Trichothecium*	*roseum*
Fungi	Ascomycota	Dothideomycetes	Mycosphaerellales	Mycosphaerellaceae	*Zymoseptoria*	*tritici*

2. Expected proportion of Fungi in balanced and unbalanced fungal mock communities

**Genus**	**Expected proportion in balanced mock community (%)**	**Expected proportion in unbalanced mock community (%)**
Chaetomium	7.69	0.50
Colletotrichum	7.69	6.17
Epicoccum	7.69	1.00
Fusarium	7.69	6.17
Microdochium	7.69	6.17
Oculimacula	7.69	20.00
Phyllosticta	7.69	0.50
Globisporangium	7.69	6.17
Ramularia	7.69	1.00
Rhizoctonia	7.69	6.17
Trichoderma	7.69	20.00
Trichothecium	7.69	6.17
Zymoseptoria	7.69	20.00

3. DNA extraction protocols

Since fungal DNA extracts were provided by different laboratories, two extraction methods were used:

Species	Protocol used
Chaetomium globosum	1
Colletotrichum coccodes	2
Epicoccum nigrum	1
Fusarium graminearum	1
Fusarium poae	1
Microdochium spp.	2
Oculimacula yallundae	2
Phyllosticta citricarpa	1
Globisporangium ultimum	2
Ramularia collo-cygni	2
Rhizoctonia solani	2
Trichoderma viride	2
Trichothecium roseum	1
Zymoseptoria tritici	2

Protocol 1:

Subcultures were performed on cellophane membranes placed on PDA medium in Petri dishes and incubated at 20–22°C. After an incubation period of 7 to 10 days, depending on the strain, the mycelium was harvested for DNA extraction using the NucleoSpin Plant kit (Macherey-Nagel). The harvested mycelium was transferred to a 2 mL tube containing stainless steel grinding beads (5 mm) and homogenized using a Retsch grinder (1 min, 30 Hz). DNA extraction was carried out following the manufacturer's protocol, and the DNA was eluted in the kit's elution buffer. The DNA concentrations were measured using a spectrophotometer, and the samples were stored at -20°C.

Protocol 2:

1. Transfer the sample into the FastPrep tube containing 100 mg of zirconium beads (0.5 mm) and add 500 µl of CTAB buffer supplemented with 5 µl of β-mercaptoethanol and 0.5 µl of Proteinase K (20 mg/ml).
2. Disrupt the sample using the FastPrep device for 40 seconds at speed 6.
3. Incubate at 42°C for 10 minutes with inversion, then at 60°C for 10 minutes with inversion.
4. Add 500 µl of chloroform:isoamyl alcohol (24:1), mix thoroughly by vortexing, incubate on ice for 10 min, and centrifuge at 13,000 rpm for 10 minutes.

5. Transfer the upper aqueous phase (~500 µl) to a new 1.5 ml tube.
6. Add 194 µl of 30% PEG and 100 µl of 5M NaCl, invert the tube five times, and centrifuge again at 13,000 rpm for 15 minutes.
7. Remove the supernatant carefully, rinse the pellet with 100 µl of 70% ethanol, and eliminate residual ethanol.
8. Dissolve the DNA pellet in 30 µl of nuclease-free water for downstream applications.
