# Supplementary figures and images for: Improving the profiling of wheat bacterial and fungal endophytic communities—a PCR clamping approach
Source: Front Microbiol. 2025 Oct 31;16:1690976. doi: 10.3389/fmicb.2025.1690976 (PMC12615186; doi:10.3389/fmicb.2025.1690976)

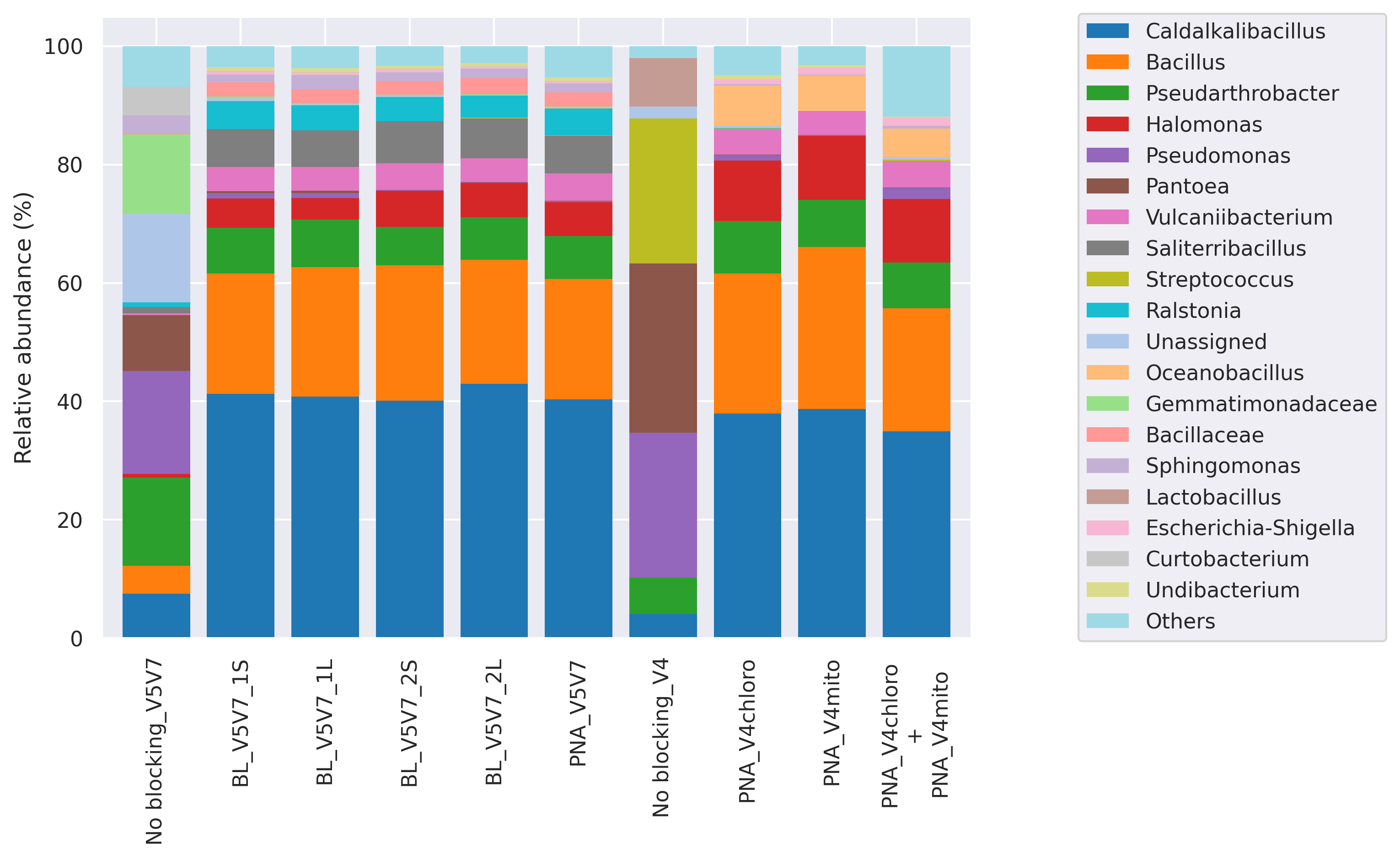

Supplement: Supplementary file 9 [file Image_1.PNG]

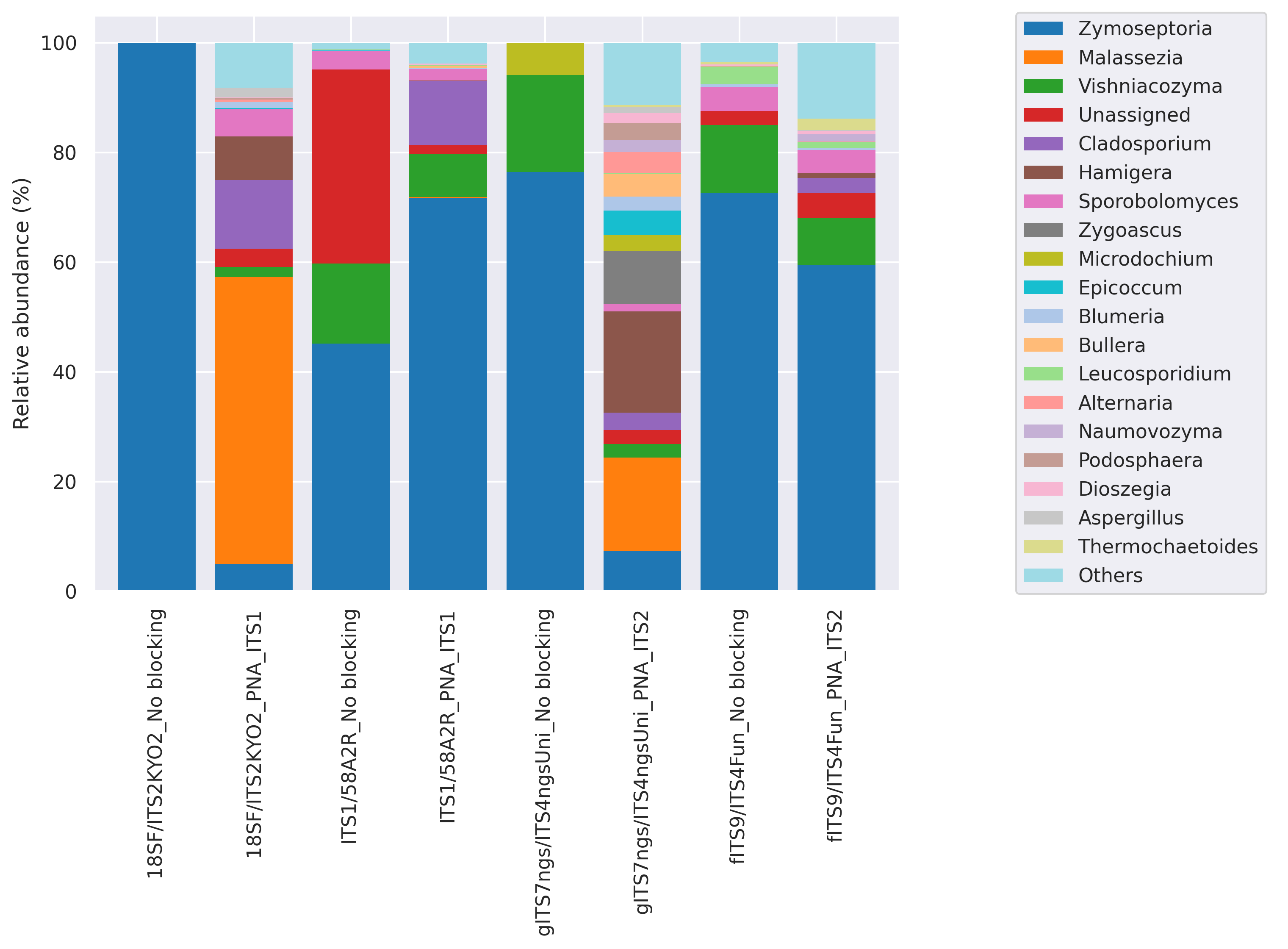

Supplement: Supplementary file 10 [file Image_2.PNG]

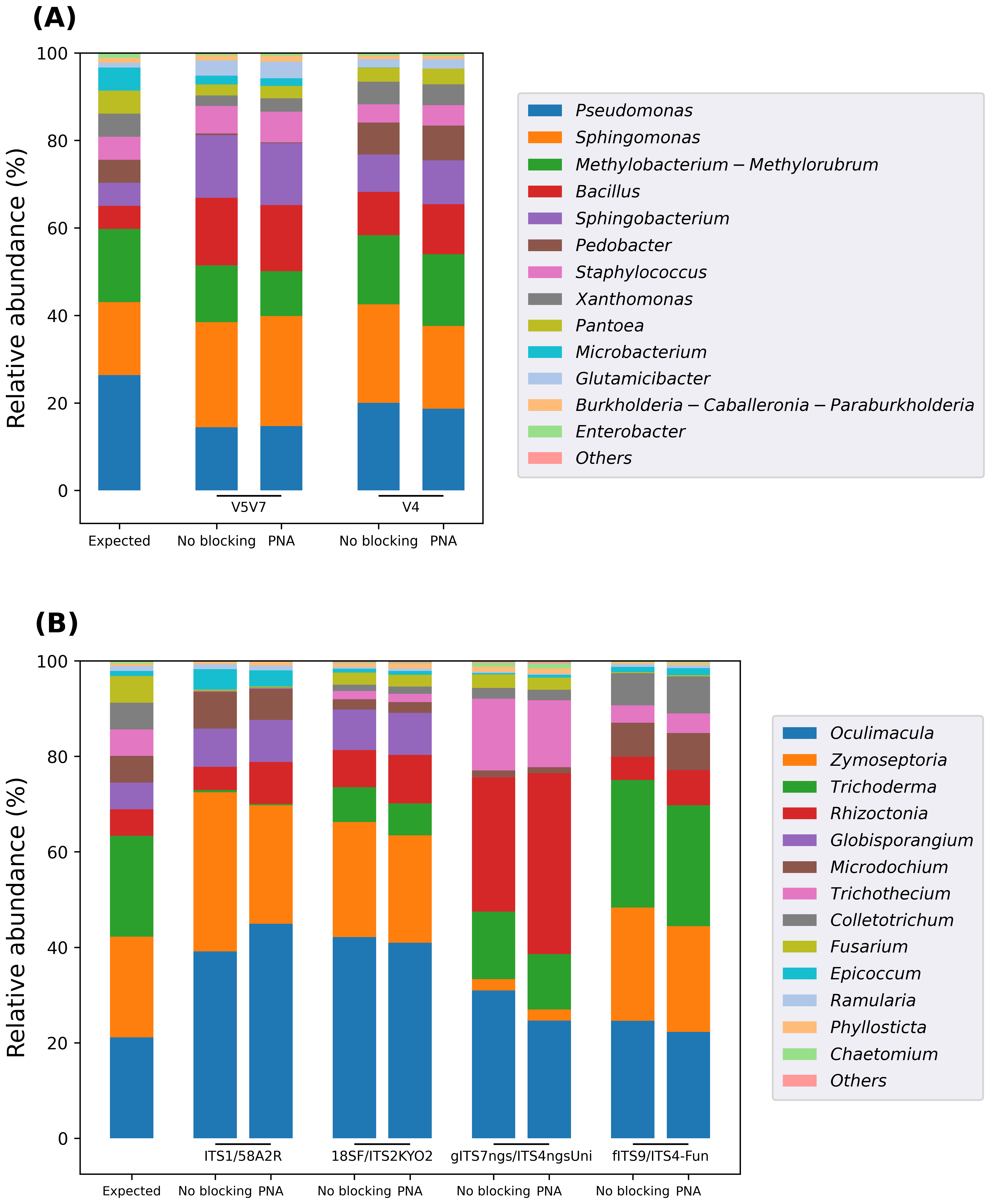

Supplement: Supplementary file 11 [file Image_3.TIF]
